# NUMB as a Therapeutic Target for Melanoma

**DOI:** 10.1016/j.jid.2021.11.027

**Published:** 2021-12-07

**Authors:** Denitsa M. Hristova, Takeshi Fukumoto, Chihiro Takemori, Le Gao, Xia Hua, Joshua X. Wang, Ling Li, Marilda Beqiri, Andrea Watters, Adina Vultur, Yusra Gimie, Vito Rebecca, Anastasia Samarkina, Haruki Jimbo, Chikako Nishigori, Jie Zhang, Chaoran Cheng, Zhi Wei, Rajasekharan Somasundaram, Mizuho Fukunaga-Kalabis, Meenhard Herlyn

**Affiliations:** 1 The Wistar Institute, Philadelphia, Pennsylvania, USA; 2 Division of Dermatology, Department of Internal Related, Kobe University Graduate School of Medicine, Kobe, Japan; 3 Department of Computer Science, New Jersey Institute of Technology, Newark, New Jersey, USA; 4 These authors contributed equally to this work.

## Abstract

The upregulation of the adaptor protein NUMB triggers melanocytic differentiation from multipotent skin stem cells, which share many properties with aggressive melanoma cells. Although NUMB acts as a tumor suppressor in various human cancer types, little is known about its role in melanoma. In this study, we investigated the role of NUMB in melanoma progression and its regulatory mechanism. Analysis of The Cancer Genome Atlas melanoma datasets revealed that high NUMB expression in melanoma tissues correlates with improved patient survival. Moreover, NUMB expression is downregulated in metastatic melanoma cells. NUMB knockdown significantly increased the invasion potential of melanoma cells in a three-dimensional collagen matrix in vitro and in the lungs of a mouse model in vivo; it also significantly upregulated the expression of the NOTCH target gene CCNE. Previous studies suggested that Wnt signaling increases NUMB expression. By mimicking Wnt stimulation through glycogen synthase kinase-3 inhibition, we increased NUMB expression in melanoma cells. Furthermore, a glycogen synthase kinase-3 inhibitor reduced the invasion of melanoma cells in a NUMB- dependent manner. Together, our results suggest that NUMB suppresses invasion and metastasis in mela- noma, potentially through its regulation of the NOTCH–CCNE axis and that the inhibitors that upregulate NUMB can exert therapeutic effects in melanoma.

## INTRODUCTION

Melanoma is a cancer that develops in melanocytes, and the phenotype of advanced-stage melanoma often resembles neural crest precursors, which are the cellular origin of me- lanocytes in the skin (Li et al., 2010). Most early-stage mel- anomas localize at the epidermal–dermal interface where normal melanocytes are located. In contrast, advanced met- astatic melanoma cells can migrate throughout tissues in a manner similar to that of neural crest cells during vertebrate embryonic development. Furthermore, the molecular signaling pathways critical for normal embryonic develop- ment and stem cell maintenance are often reactivated in melanoma cells (Liu et al., 2014). One of them is the NOTCH pathway, which has been implicated in melanoma patho- genesis and in the regulation of adult stem cells. We previ- ously showed that NOTCH plays a role in the self-renewal of multipotent, neural crest–like skin precursors, which is a potential reservoir for skin melanocytes (Fukunaga-Kalabis et al., 2015). Melanocyte differentiation from multipotent precursor cells is stimulated when the precursors are acti- vated by canonical Wnt ligands in combination with mito- gens such as stem cell factor and endothelin. The initial step of this differentiation is triggered by upregulated NUMB, which inhibits NOTCH signaling in skin precursors (Fukunaga-Kalabis et al., 2015).

NUMB was identified as a cell-fate determinant in Drosophila embryos, which was later confirmed in verte- brates (Uemura et al., 1989; Zhong et al., 1996). In neuro- genesis, NUMB binds to the active form of intracellular NOTCH and inhibits NOTCH-dependent gene expression to repress neuronal differentiation (Wakamatsu et al., 1999). In addition to its role in NOTCH inhibition, NUMB induces p53 expression and it inhibits the Hedgehog signaling pathway, also important players in aggressive melanoma (Colaluca et al., 2008; Di Marcotullio et al., 2006). NUMB has been suggested to suppress tumors in several cancers, where NUMB expression is frequently downregulated in malignant cells (Pece et al., 2011). In melanoma, NUMB is a target of microRNA-146a, which has an oncogenic role (Forloni et al., 2014). Moreover, NUMB is required for the stabilization and localization of the cell cycle regulator PLK1, suggesting that dysregulation of NUMB expression can contribute to melanoma development through mitotic errors (Schmit et al., 2012). Because metastatic melanoma cells share embryonic and migratory phenotypes with neural crest–like precursors in the skin, we hypothesized that dysregulation of NUMB is involved not only in melanoma development but also in its progression. In this study, we investigated the biological, mechanistic, and therapeutic role of NUMB in melanoma.

## RESULTS

### NUMB expression correlates with melanoma survival and is downregulated in metastatic melanoma cells

**Figure 1.**
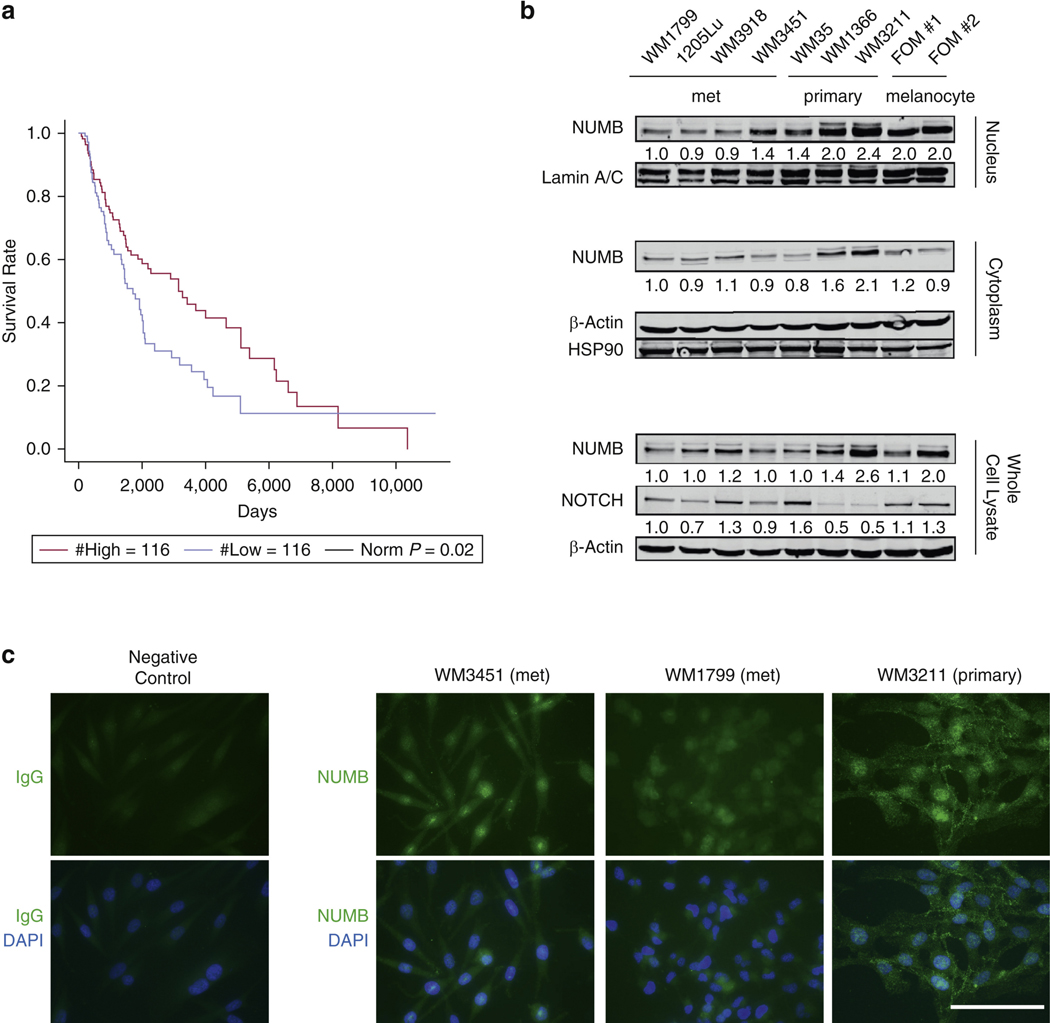
Low *NUMB* levels are correlated with decreased overall survival in patients with melanoma. (a) KaplaneMeier survival curve analysis of the overall survival of patients with low and high *NUMB* mRNA expression in melanoma was used to compare a quarter each of the patients having the highest with those having the lowest *NUMB* expression in their tumor (Cancer Genome Atlas Network, 2015). (b) Immunoblot analysis showing NUMB and NOTCH expression in melanocytes (FOM#1 and #2), primary melanoma cell lines (WM35, WM1366, WM3211), and met melanoma cell lines (WM1799, 1205Lu, WM3918, WM3451) in whole-cell lysates, cytoplasmic fractions, or nuclear fractions. Blotting for b-actin, HSP90, and lamin A/C serves as loading controls. The relative intensities of the immunoblot bands were quantified. (c) Immunofluorescent staining showing NUMB expression in melanoma cell lines. Bar = 100 mm. met, metastatic.

First, to investigate the potential involvement of NUMB in melanoma progression, we analyzed melanoma RNA- sequencing data from The Cancer Genome Atlas, together with the patients’ clinical information (Cancer Genome Atlas Network, 2015). KaplaneMeier analyses showed that patients with lower *NUMB* gene expression had significantly decreased overall survival (log-rank test, *P* = 0.02) (Figure 1a). Recent evidence showed the pivotal role of long noncoding RNAs (lncRNAs) in uveal melanoma by regulating proliferation, invasion, and metastasis (Cheng et al., 2016; Milán-Rois et al., 2021). Thus, a second analysis was focused on NUMB lncRNA, which was marginally associated with patient survival (*P* = 0.04, for both log-rank test and the Cox regression) in the 232 The Cancer Genome Atlas samples. To see whether the association of NUMB lncRNA with patient survival is an independent and driving signal or whether it is driven by the correlation between NUMB and NUMB lncRNA, we performed Cox regression with both NUMB and NUMB lncRNA as covariates. Inter- estingly, we observed that NUMB remained significant (*P* = 0.04), whereas NUMB lncRNA was no longer significant (*P* = 0.25), suggesting that it is NUMB that drives the survival association.

NUMB protein expression was downregulated in three of four metastatic melanoma cell lines in the nucleus. In contrast, two of the three examined primary melanoma cell lines demonstrated similar levels of NUMB expression to those of normal melanocytes (Figure 1b). These results are consistent with those of a previous study showing that the expression of NUMB is reduced in cancer tissues compared with that in normal tissues, with the reduction even worse in tumor tissues with TNM stages III–IV than in those with stages I–II (Liang et al., 2019). Immunofluorescent staining showed that in the human melanoma cell lines WM1799, WM3451, and WM3211, NUMB was expressed mainly in the nucleus but also in the cytoplasm and cell membrane (Figure 1c). These data suggest that NUMB may function both inside and outside the nucleus.

Previous studies suggested that the activation of NOTCH signaling is necessary for melanoma progression (Aydin et al., 2014; Balint et al., 2005; Liu et al., 2006). Consis- tent with previous reports, we observed that NOTCH was barely expressed in WM1366 and WM3211 melanoma cells that have a high NUMB expression (Figure 1b) (Choi et al., 2021; Li et al., 2019; Wakamatsu et al., 1999).

### Depletion of NUMB increases melanoma invasion and metastasis

**Figure 2.**
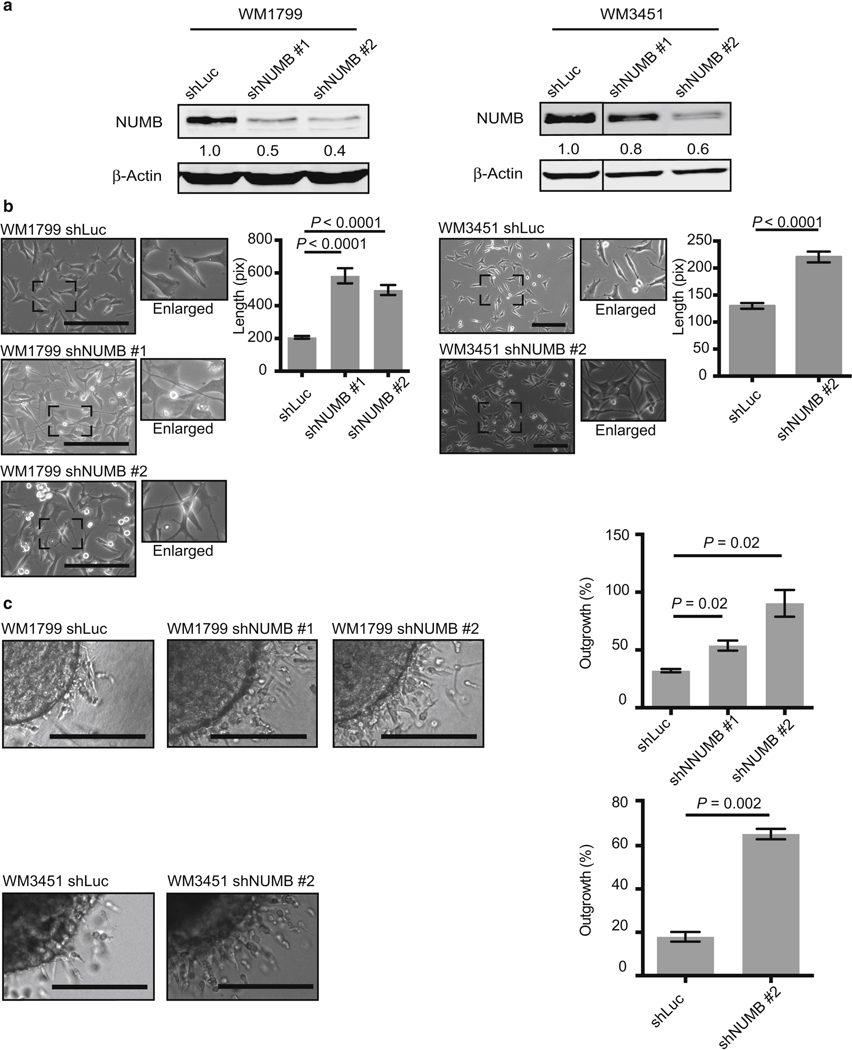
Knockdown of *NUMB* promotes cell invasion in metastatic melanoma cell lines. (a) Immunoblot analysis showing NUMB expression in metastatic melanoma cell lines with a control vector (shLuc) or shNUMB #1 and #2. Blotting for b-actin serves as a loading control. The relative intensities of the immunoblot bands were quantified. (b) Downregulation of *NUMB* by shRNA in metastatic melanoma cell lines leads to morphological changes, which were quantified. Bar = 200 mm. (c) shNUMB-transduced cells or control vector–transduced cells were grown as spheroids embedded in a 3D collagen matrix to test their invasive ability. *NUMB* knockdown cell lines showed increased invasion in WM1799 (on day 1) and WM3451 (on day 2) melanoma cell lines compared with that in the control cell lines. Bar = 200 mm. 3D, three-dimensional; pix, pixel; shLuc, short hairpin RNA targeting firefly luciferase; shNUMB, short hairpin RNA targeting *NUMB*; shRNA, short hairpin RNA.

To assess whether NUMB downregulation is directly involved in melanoma aggressive behavior, we performed *NUMB* knockdown (KD) experiments. The metastatic melanoma cell lines WM1799 and WM3451 were transfected with lentiviral vectors encoding short hairpin RNAs targeting *NUMB* (shNUMBs) to downregulate NUMB expression (Figure 2a and Supplementary Figure S1a). *NUMB* KD caused morphological changes in the melanoma cells: they became spindle shaped with significantly elongated dendrites resembling mesenchymal cells (Figure 2b and Supplementary Figure S2). *NUMB* KD did not affect the proliferation of WM1799 and WM3451 cells over a 6-day period (Supplementary Figure S1b). To explore whether NUMB af- fects melanoma invasion, we employed a three-dimensional spheroid model, which represents the complexity and het- erogeneity of tumors within tissues (Smalley et al., 2006). *NUMB* KD induced a significant increase in the invasion of melanoma spheroids into collagen matrices (Figure 2c and Supplementary Figure S3a and b). Similar experiments were also performed with the primary melanoma cell line WM35 (Supplementary Figures S2 and S3). *NUMB* KD induced a significant increase in the invasion of melanoma spheroids into collagen matrices, although *NUMB* KD did not affect the proliferation over a 7-day period (Supplementary Figures S2c and S3c).

**Figure 3.**
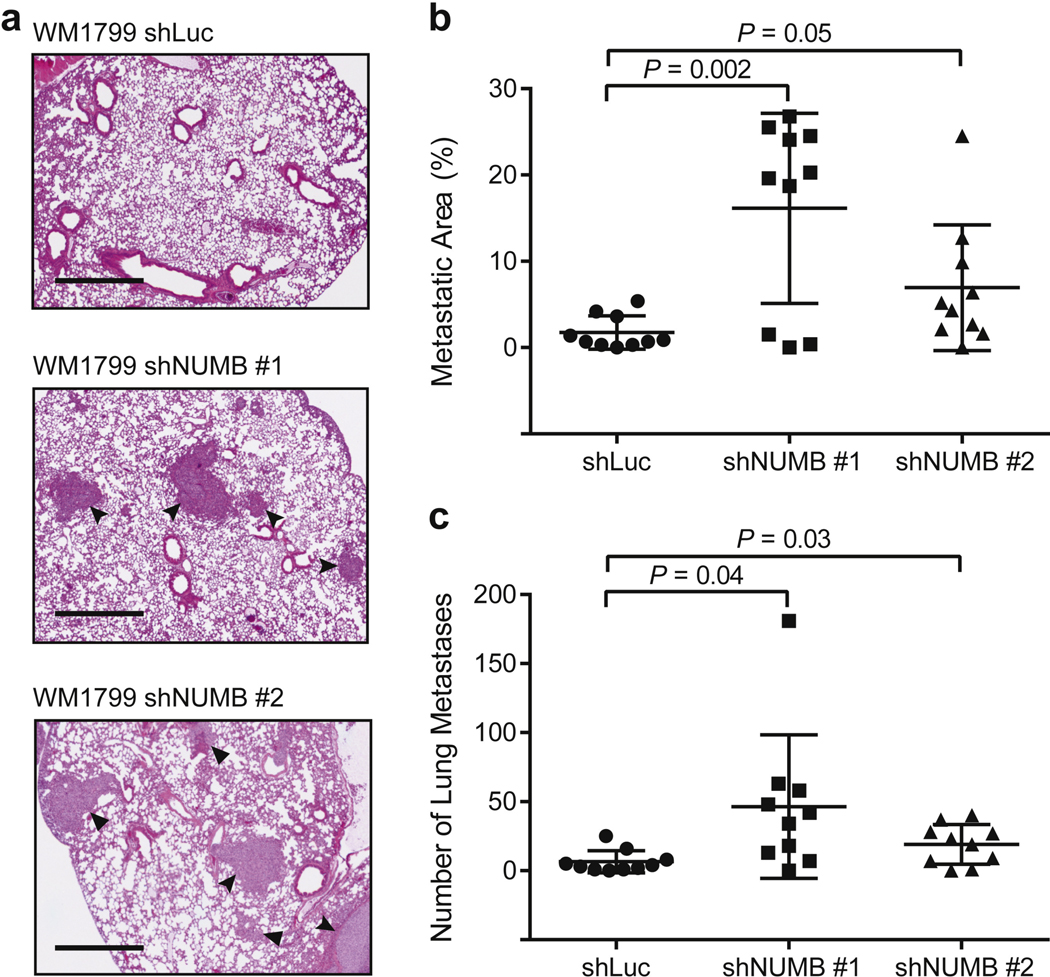
Knockdown of *NUMB* promotes melanoma metastasis in vivo. (a) H&E staining of lung tissue sections showing metastatic lesions in in vivo metastasis assays. Mice were injected intravenously with WM1799 melanoma cells transduced with a control shRNA or with shRNAs targeting *NUMB*. Arrowheads indicate metastasis. Bar = 1 mm. (b) Quantification of the percentage of metastatic area per lung tissue section (n = 10). (c) Quantification of the number of lung metastases per mouse lung section (n = 10). shLuc, short hairpin RNA targeting firefly luciferase; shRNA, short hairpin RNA.

Next, we sought to investigate whether *NUMB* KD pro- motes the progression of melanoma in vivo. We tested lung colony formation in an experimental metastasis model by injecting WM1799 melanoma cells intravenously into NOD/SCID IL2Rg^null^ or NSG mice. After 9 weeks, the mice were euthanized, and lung samples were collected for the detection of metastases. Both the area and the number of metastatic colonies developed in the lungs were found to be increased in mice injected with WM1799 shNUMB cells compared with those found in the WM1799 short hairpin RNA targeting firefly luciferase control cell–injected group (Figure 3). These data suggest that NUMB plays an important role in the invasion and metastatic potential of melanoma.

### NUMB is induced by the inhibition of glycogen synthase kinase-3 in melanoma cells

**Figure 4.**
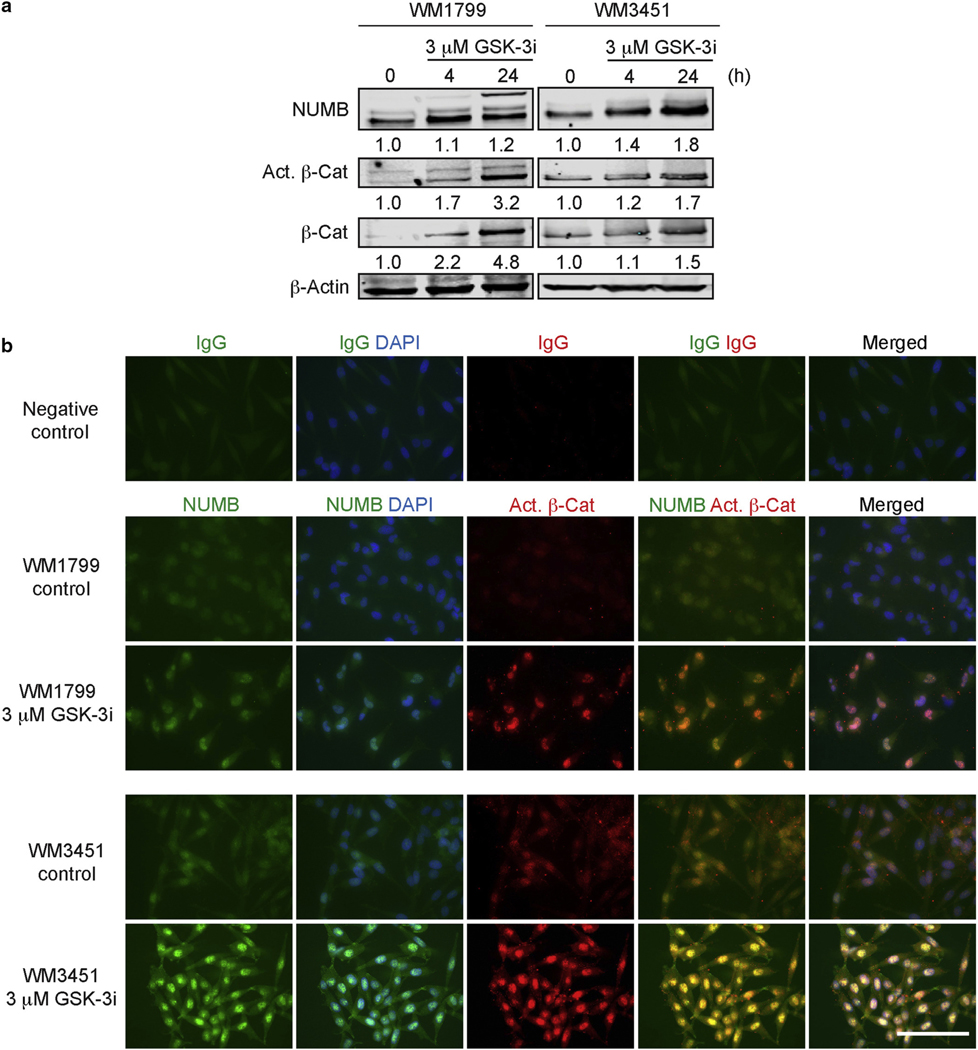
Inhibition of GSK-3 upregulates NUMB in metastatic melanoma cell lines. (a) Immunoblot analysis showing the expression of NUMB, active b-Cat, and total b-Cat in WM1799 and WM3451 melanoma cell lines treated with GSK-3i IX. The 0 h–treated samples refer to the cells incubated for 24 h in the media without GSK-3i IX. Blotting for b-actin serves as a loading control. The relative intensities of the immunoblot bands were quantified. (b) Immunofluorescent staining showing the expression of NUMB and active b-catenin after treatment with GSK-3i IX in WM1799 and WM3451 melanoma cell lines. Bar = 100 mm. b- Cat, b-catenin; Act., active; GSK-3, glycogen synthase kinase-3; GSK-3i, glycogen synthase kinase-3 inhibitor; h, hour.

Our KD experiments suggested that NUMB negatively reg- ulates melanoma aggressive behavior. We previously showed that NUMB expression is increased by canonical Wnt ligands (Fukunaga-Kalabis et al., 2015). In this study, we sought to test whether NUMB expression could be increased in melanoma cells by pharmacologically acti- vating the canonical Wnt pathway. To do so, we blocked glycogen synthase kinase-3 (GSK-3), which is a negative regulator of canonical Wnt signaling, using the GSK-3 inhibitor IX. This inhibitor upregulated the expression of NUMB protein in melanoma cell lines and activated b- catenin (Figure 4a). Activation of b-catenin was also confirmed by the upregulation of the mRNA expression of its target gene, *AXIN2* (Supplementary Figure S4). Further- more, upregulated NUMB was not only found in the cytoplasm but was also colocalized with active b-catenin in the nuclei of WM1799 and WM3451 cells (Figure 4b). These results were consistent with those of previous studies reporting NUMB interaction with b-catenin (Colaluca et al., 2018; Liang et al., 2019).

### GSK-3 inhibition decreases melanoma invasion in a NUMB- dependent manner

**Figure 5.**
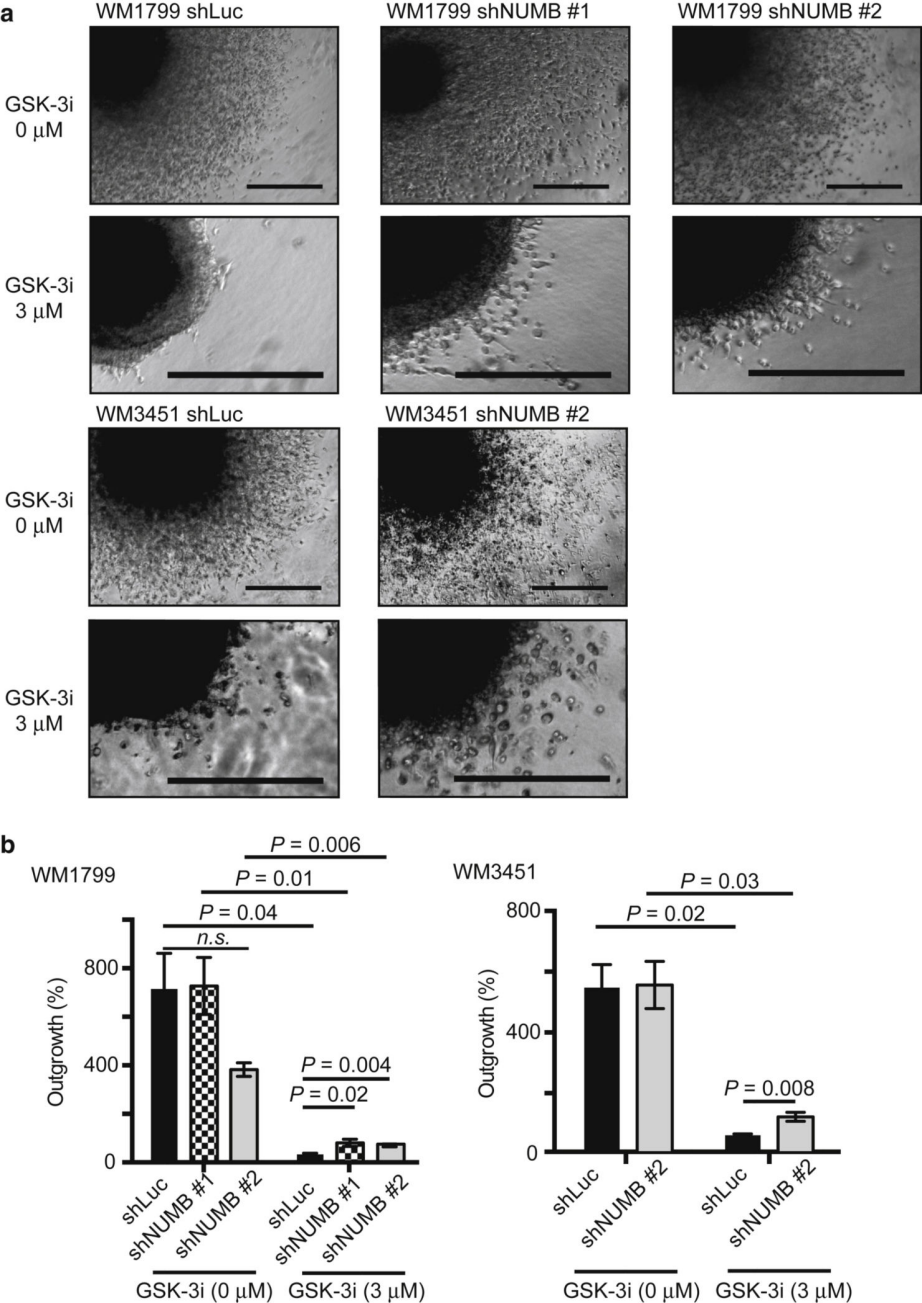
Knockdown of *NUMB* rescues cell invasion in melanoma cells treated with GSK-3i. (a, b) Control WM1799 cells (WM1799 shLuc) or WM1799 cells transduced with shNUMB (WM1799 shNUMB #1 and #2) were grown as spheroids, embedded in 3D collagen gel, and allowed to invade with or without GSK-3i IX at 3 mM for 11 days. Control WM3451 cells (WM3451 shLuc) or WM3451 cells transduced with shNUMB (WM3451 shNUMB #2) were grown as spheroids, embedded in 3D collagen gel, and allowed to invade with or without the presence of GSK-3i IX at 3 mM for 8 days. Bars = 200 mm. 3D, three-dimensional; GSK- 3i, glycogen synthase kinase-3 inhibitor; n.s., not significant; shLuc, short hairpin RNA targeting firefly luciferase; shNUMB, short hairpin RNA targeting *NUMB*.

Previous studies have shown that GSK-3 promotes cell sur- vival, growth, motility, and drug resistance in melanoma cells (Atkinson et al., 2015; John et al., 2012; Kubic et al., 2012; Panka et al., 2008; Smalley et al., 2007; Zimmerman et al., 2013). We sought to investigate the preventive efficacy of GSK-3 inhibitors in melanoma. In the untreated spheroids, the difference in invasion between control and *NUMB* KD was less obvious after >1 week owing to the high rate of invasion and model saturation (Figure 5). However, treatment with 3 mM GSK-3 inhibitor IX was sufficient to inhibit the invasion of three-dimensional melanoma spheroids into collagen matrices (Figure 5 and Supplementary Figure S5). This dose did not kill the melanoma cells, as evident from their recurrent invasion after drug withdrawal (Figure 5 and Supplementary Figure S6). Notably, *NUMB* KD reduced the anti-invasive effects of the GSK-3 inhibitor IX (Figure 5). These data suggest that GSK-3 inhibition suppresses melanoma invasion in a NUMB- dependent manner.

### The regulation of NUMB in invasion activity through the cell cycle

To investigate the potential mechanism by which NUMB regulates invasion in melanoma, we performed quantitative real time-PCR to evaluate the expression of *CCNE* and *MITF* in WM1799 and WM3451. *CCNE* and *MITF* regulate the cell cycle, cell survival, and invasion and have also been reported to be regulated by the *NUMB–NOTCH* axis (Li et al., 2014; Schmit et al., 2012; Wu et al., 2014; Zender et al., 2013).

**Figure 6.**
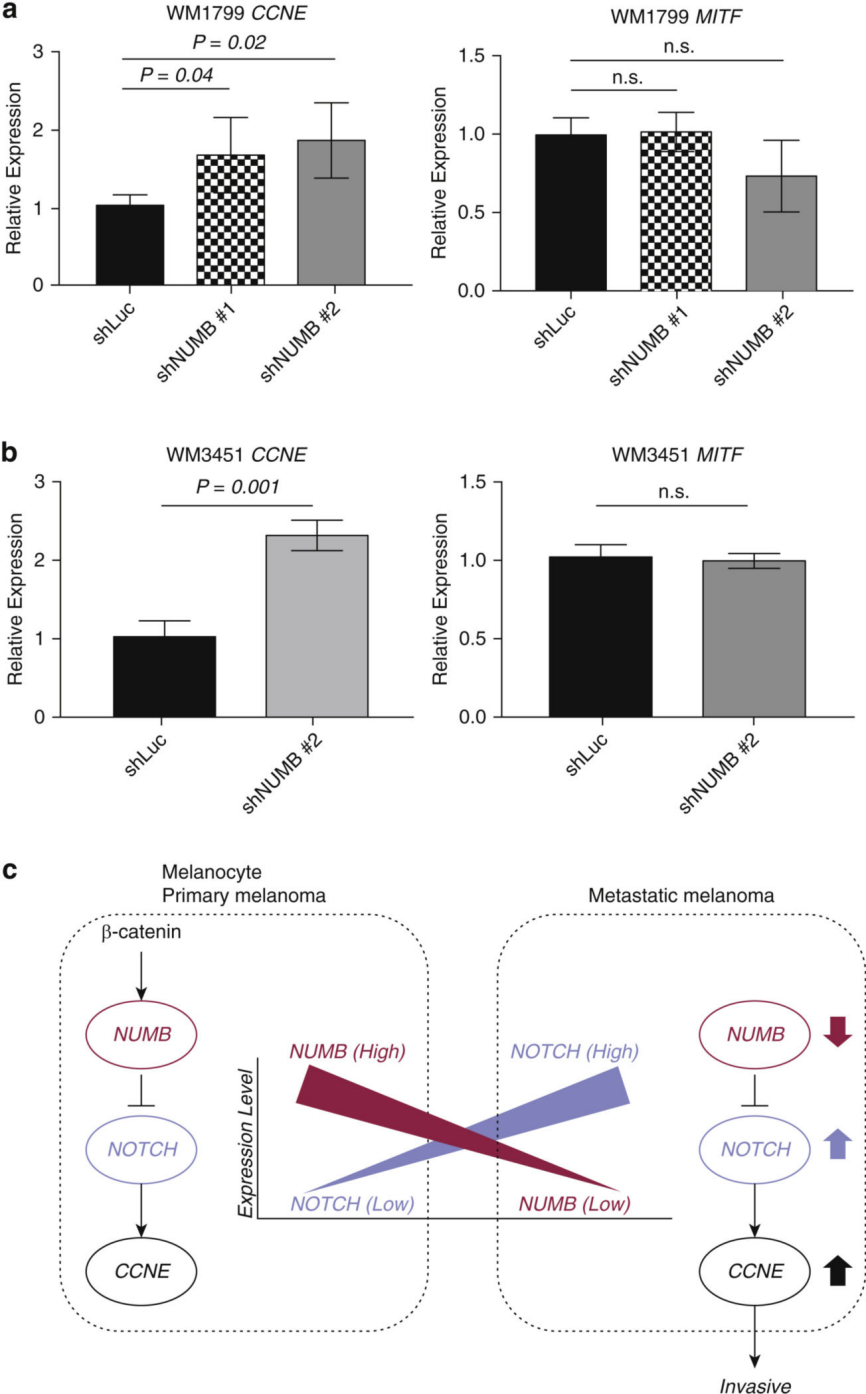
The effects of NUMB knockdown on CCNE and MITF expression. (a, b) Quantitative real time-PCR showing the expression of CCNE and MITF in the metastatic melanoma cell lines (a) WM1799 and (b) WM3451 transduced with shNUMB #1 and #2 compared with that in shLucs. mRNA levels of CCNE and MITF were normalized to that of GAPDH. (c) The potential mechanism of NUMB to regulate invasion activity in melanocyte and melanoma through the NUMB–NOTCH–CCNE axis. n.s., not significant; shLuc, short hairpin RNA targeting firefly luciferase; shNUMB, short hairpin RNA targeting NUMB.

*NUMB* KD increased the expression of *CCNE* in WM1799 and WM3451, whereas *NUMB* KD did not affect the expression of *MITF* (Figure 6a and b). The regulation of NUMB in invasion may thus be dependent on the cell cycle (Figure 6), which is consistent with previous reports showing the cell cycle–dependent regulation of NUMB in invasion activity (Li et al., 2014; Schmit et al., 2012; Wu et al., 2014; Zender et al., 2013).

## DISCUSSION

In this study, we investigated the role of NUMB in the aggressive behavior of melanoma and its associated molec- ular mechanisms. NUMB is highly expressed in normal melanocytes and primary melanoma cells, whereas its expression is decreased in metastatic, aggressive melanoma cells. The analysis of The Cancer Genome Atlas dataset revealed that low NUMB expression was associated with poor overall survival. Through loss-of-function experiments, we show that NUMB downregulation leads to increased cell invasion and high metastatic capability. Furthermore, NUMB downregulation increased the expression of the *NOTCH* target gene *CCNE*, which is implicated in melanoma invasion and metastasis (Bales et al., 2005). In terms of treatment for melanoma, immune checkpoint inhibitors play a crucial role by dramatically improving the treatment outcome of malignancies. However, the response rates of immune checkpoint inhibitors against melanoma are insufficient, and new therapeutic strategies now need to be identified for patients with melanoma (Fukumoto et al., 2021). Melanoma cells and neural crest–like skin pre- cursors share many biological properties, such as migratory ability, self-renewal capability, and the expression of neural crest markers. Recent studies suggest that targeting neural crest–specific pathways provides an untapped reservoir of novel options (Shakhova et al., 2012; White et al., 2011). NUMB is an adaptor protein that interferes with the NOTCH pathway and has been characterized as a fate determinant during the development of an organism (Cohen and Hyman, 1994). Our previous study identified NUMB as a critical molecule in melanocyte differentiation from neural crest–like skin precursors (Fukunaga-Kalabis et al., 2015). Meanwhile, the self-renewal of neural crest–like skin precursors was shown to be controlled by NOTCH activation. Bringing these molecular players all together, the canonical Wnt stimulation was shown to upregulate NUMB, thereby inhibiting the NOTCH pathway. The suppression of NOTCH signaling reportedly triggers the exit from self-renewal circuitry and promotes melanocyte dif- ferentiation. Such NUMB induction and consequent repression of NOTCH signaling by canonical Wnt ligands have also been reported in chick somitic myogenesis (Holowacz et al., 2006). In melanoma, the NOTCH pathway plays pivotal roles in the development, growth, survival, and progression of tumor cells (Bedogni, 2014). Among several NOTCH inhibitory agents, g-secretase inhibitors have shown antitumor effects in preclinical in vivo models. However, clinical trials using these agents have been hampered by substantial toxicities at higher doses required for inhibiting the NOTCH pathway in tumors, when used as single agents (Takebe et al., 2014). Because many mechanisms contribute to NOTCH activation, the effective targeting of the pathway requires a more detailed understanding of pathway regulation and in a context-specific manner (Rizzo et al., 2008). In addition, the associated molecular players and pathways have not yet been fully investigated in melanoma and may provide encouraging results in the clinics.

Importantly, our study shows that NUMB expression can be increased by directly inhibiting a serine/threonine protein kinase, GSK-3, using a small-molecule compound. Aberrant GSK-3 signaling has been reported in numerous cancer types, and GSK-3 is highly expressed in melanoma tissues (John et al., 2012; Madhunapantula et al., 2013). In preclinical melanoma models, the inhibition of GSK-3 decreased cell proliferation, reduced migration and metastasis, and enhanced apoptosis (Atkinson et al., 2015; John et al., 2012; Kubic et al., 2012; Panka et al., 2008; Smalley et al., 2007; Zimmerman et al., 2013). Our data now show that NUMB is a pivotal mediator in the suppression of invasion by GSK-3 inhibitors. Depletion of NUMB can lead to the increased expression of NOTCH target genes, which are implicated as therapeutic targets in the invasion of cancer cells (Binda et al., 2017; Tsuru et al., 2015; Weeraratna et al., 2002).

Emerging evidence shows that NUMB participates in invasion and proliferation, but the details remain controversial. Some studies reported that NUMB inhibits invasion and proliferation (Li et al., 2019; Liang et al., 2019); other conflicting studies reported that NUMB promotes cell growth (Schmit et al., 2012; Wu et al., 2014). The conclusions may depend on the variety of NUMB isoforms present or the types of cell lines or specific stages of cell development, and further studies are needed to elucidate the complexity of NUMB regulation in different biological settings (Choi et al., 2021; Colaluca et al., 2018; Liang et al., 2019; Schmit et al., 2012; Wu et al., 2014). NUMB negatively regulates the *NOTCH* target gene *CCNE* (Li et al., 2014; Zender et al., 2013). Importantly, *CCNE* has been reported to be critical for pro- gression of cancers, including melanoma (Bales et al., 2005). Together with our current findings, we suggest that NUMB suppresses invasion and metastasis in melanoma potentially through the *NUMB–NOTCH–CCNE* axis that regulates the cell cycle (Figure 6c). Furthermore, the target-specific inhibitors that can upregulate NUMB such as GSK-3 may exert useful therapeutic effects in aggressive melanomas.

One limitation is that the mechanism by which GSK-3 inhibition increases NUMB levels remains to be elucidated.

Previous studies suggested that GSK-3 targets multiple sub- strates in addition to negatively regulating b-catenin signaling (Sutherland, 2011). For example, the MDM2 oncoprotein is one of the targets of GSK-3; therefore, GSK-3 inhibition leads to the hypophosphorylation of MDM2 and p53 stabilization (Kulikov et al., 2005). Interestingly, MDM2 mediates the ubiquitination and degradation of the NUMB protein (Yogosawa et al., 2003). Because MDM2 overexpression is an independent predictor of survival in patients with melanoma (Polsky et al., 2002), it is possible that GSK-3 regulates NUMB expression in an MDM2-dependent manner.

## MATERIALS AND METHODS

### Cell culture and reagents

Melanoma cell lines were established from melanoma specimens that were obtained in accordance with consent procedures approved by the Internal Review Boards of the Perelman School of Medicine at the University of Pennsylvania (Philadelphia, PA) and The Wistar Institute (Philadelphia, PA). Human foreskin specimens were obtained from the Cooperative Human Tissue Network (http://www.chtn.nci.nih.gov) Written informed consent was obtained when collecting tissues from patients according to the policies and pro- cedures of the Cooperative Human Tissue Network, complying with federal human subjects regulations. The Wistar institutional review board approved foreskin sample collection from the Cooperative Human Tissue Network.

Human melanoma cells (WM1799, 1205Lu, WM3918, WM3451, WM35, WM1366, and WM3211) were isolated as previously described and cultured in 2% Mel (Satyamoorthy et al., 1997) or in melanocyte differentiation media (Li et al., 2010) supplemented with 20 ng/ml of recombinant Wnt3a or Wnt7a (R&D Systems, Minne- apolis, MN). Human primary melanocytes were isolated from neonatal foreskins as previously described (Hsu et al., 2005) and cultured in 254CF media (Life Technologies, Carlsbad, CA). Lentiviral particles were produced in human embryonic kidney 293T cells, which were cultured in DMEM (Life Technologies) supple- mented with 10% fetal bovine serum. The GSK-3 inhibitor IX was purchased from Calbiochem (EMD Millipore, Billerica, MA).

### Lentiviral constructs

Lentiviral constructs encoding small hairpin RNA sequences target- ing NUMB were purchased from GE Dharmacon (Lafayette, CO) (oligo identifications: TRCN 0000007227 and TRCN0000007226, referred to as shNUMB_1 and shNUMB_2, respectively). A lentiviral construct encoding small hairpin RNA sequences targeting lucif- erase (SHC007, Sigma-Aldrich, St. Louis, MO) served as vector control.

### Western blot analysis

Western blotting was performed as previously described elsewhere (Fukunaga-Kalabis et al., 2015). Whole-cell lysates were generated by washing the cells with PBS and lysed with radio- immunoprecipitation assay buffer. Nuclear and cytoplasmic fraction proteins were isolated using the NE-PER Nuclear Protein Extraction Kit (Thermo Fisher Scientific, Fremont, CA) following the manufac- turer’s instructions. Proteins were separated on 10% polyacrylamide gels and blotted onto polyvinylidene difluoride membranes using a Trans-Blot Turbo Transfer System (Bio-Rad Laboratories, Berkeley, CA). Primary antibodies for NUMB (catalog number ab14140, dilution 1:1,000; Abcam, Cambridge, MA), active-b-catenin, 8E7

(catalog number 05–665, dilution 1:1,000; EMD Millipore), b-catenin (catalog number 610153, dilution 1:1,000; BD Biosciences, Franklin Lakes, NJ), NOTCH1 (D6F11) XP (catalog number 4380, dilution 1:1,000; Cell Signaling Technology, Danvers, MA), b-actin (catalog number A5441, dilution 1:5,000; Sigma-Aldrich), HSP90 (C45G5) (catalog number 4877, dilution 1:1,000; Cell Signaling Technology), and lamin A/C (catalog number 2032, dilution 1:1,000; Cell Signaling Technology) were used for membrane hybridization. Signals from IRDye (LI-COR Biosciences, Lincoln, NE) secondary antibodies were detected using the LI-COR Odyssey Infrared Imag- ing System (LI-COR Biosciences).

### Quantification of morphological changes

Using ImagePro Plus software (Media Cybernetics, Rockville, MA), lines were drawn along the longest axis of the cells to measure the cell length. At least 20 cells were analyzed per condition. The length was expressed in pixels.

### Three-dimensional spheroid assays

Melanoma spheroids were prepared as previously described (Smalley et al., 2006). Briefly, 5,000 melanoma cells were plated per well into a 96-well plate coated with 1.5% agar (catalog number DF0142–15-2; BD Difco, Hampton, NH). After the cells formed three-dimensional spheroid structures, the spheroids were embedded in bovine collagen I (Organogenesis, Canton, MA) containing 9% 10 EMEM (catalog number 12–684F; Lonza, Basel, Switzerland), 0.8% 200 mM L-glutamine (catalog number 25030024; Thermo Fisher Scientific, Waltham, MA), 10% fetal bovine serum, and 1.7% sodium bicarbonate (7.5% stock, catalog number 17–613E; Thermo Fisher Scientific). Spheroids were imaged with a Nikon TE2000 inverted microscope, using a Q-Imaging Retiga EX digital camera and ImagePro Plus software. The growth of the spheroids was quantified using ImagePro Plus software (Media Cybernetics). One representative spheroid from each well was selected. Then, the spheroid core and outgrowth areas were measured in square micrometer, and the percentage of outgrowth (the value reported in the analysis) was calculated as the ratio of the spheroid outgrowth to the spheroid core.

### Quantitative real time-PCR

mRNAs from cells were collected using QIAshredder (Qiagen, Hil- den, Germany) and the RNeasy kit (Qiagen) following the manu- facturer’s instructions. cDNA was synthesized from mRNA using the Maxima First Strand cDNA Synthesis Kit (Thermo Fisher Scientific) according to the manufacturer’s instructions. Fast SYBR Green Master Mix (Applied Biosystems, Wilmington, DE) was used to perform quantitative real-time PCR on a 7500 Fast Real-Time PCR System (Applied Biosystems). The obtained values were normalized to *GAPDH* expression. The following primers for target genes were purchased from Integrated DNA Technologies (Coralville, IA): for- ward 5′-CCGGCATGCTCCAATTG-3′ and reverse 5′-TCTGGCTAA- GAGCAGGAAAACC-3′ for *NUMB*, forward 5′- CCCATCATGCCGAGGGAG-3′ and reverse 5′- TATTGTCCCAAGGCTGGCTC-3′ for *CCNE*, forward 5′- AAACCCCACCAAGTACCACA-3′ and reverse 5′- ACATGGCAAGCTCAGGAC-3′ for *MITF*, and forward 5′- CTCCTTATCGTGTGGGCAGT-3′ and reverse 5′-CTTCATCCTCTCG- GATCTGC-3′ for *AXIN2*.

### Cell proliferation assays

First, 1 10^3^ cells per well were seeded in 96 well-plates. On days 1, 4, and 6, CellTiter 96 AQueous MTS reagent (Promega, Madison, WI) was added to each well according to the manufacturer’s instructions. After 4 hours of incubation at 37 ^0^C, the absorbance was measured at 490 nm.

### Immunofluorescence staining

Cells were seeded at 4e6 10^4^ cells/well in fibronectin-coated four-well glass chamber slides in 2% Mel melanoma growth me- dia. The cells were fixed with 2% paraformaldehyde in PBS for 10 minutes at room temperature. Incubation with 0.2% Triton X-100 for 5 minutes at room temperature was used to permeabilize the cells. After blocking with 2% BSA for 30 minutes at room temperature, cells were incubated with primary antibodies specific for NUMB (catalog number ab14140, dilution 1:250; Abcam) and active b- catenin, 8E7 (catalog number 05–665, dilution 1:250; EMD Milli- pore). Alexa Fluor 488 goat anti-rabbit IgG H&L (catalog number A11034, 1:500; Life Technologies) and Alexa Fluor 555 goat anti- mouse IgG (H &L) (catalog number A21424, 1:500; Life Technolo- gies) were used as secondary antibodies. Microscopy and photo- capture were performed with a Nikon Eclipse 80i upright fluorescence microscope, using a Q Imaging EXI Aqua digital camera and ImagePro Plus 7.0 software.

### Murine experimental metastasis assay

Animal experiments were performed with the approval of the Institutional Animal Care and Use Committee of The Wistar Institute in a facility accredited by the Association for the Assessment and Accreditation of Laboratory Animal Care. Subconfluent cultures of WM1799 short hairpin RNA targeting firefly luciferase, WM1799 shNUMB #1, and WM1799 shNUMB #2 melanoma cells were harvested, washed, and resuspended in sterile PBS. Approximately 100,000 cells per mouse were injected into the tail veins of nonobese diabetic/severe combined immunodeficient IL-2 receptor-g chain null mice. Mice were killed on day 63, and the lungs were removed, washed in PBS, and fixed in 10% formalin.

## Supplementary Material

Figure S1

Figure S2

Figure S3

Figure S5

Figure S4

Figure S6

Suppl Figure Legend

## Data Availability

All data are available in the main text or in the Supplementary Materials and Methods.

## Abbreviations:

GSK-3: glycogen synthase kinase-3
KD: knockdown
lncRNA: long noncoding RNA
shNUMB: short hairpin RNA targeting NUMB
