# Supplementary figures and images for: NUMB as a Therapeutic Target for Melanoma
Source: J Invest Dermatol. Author manuscript; Available in PMC 2022 Nov 28. (PMC9704357; doi:10.1016/j.jid.2021.11.027)

**a**WM1799 *NUMB*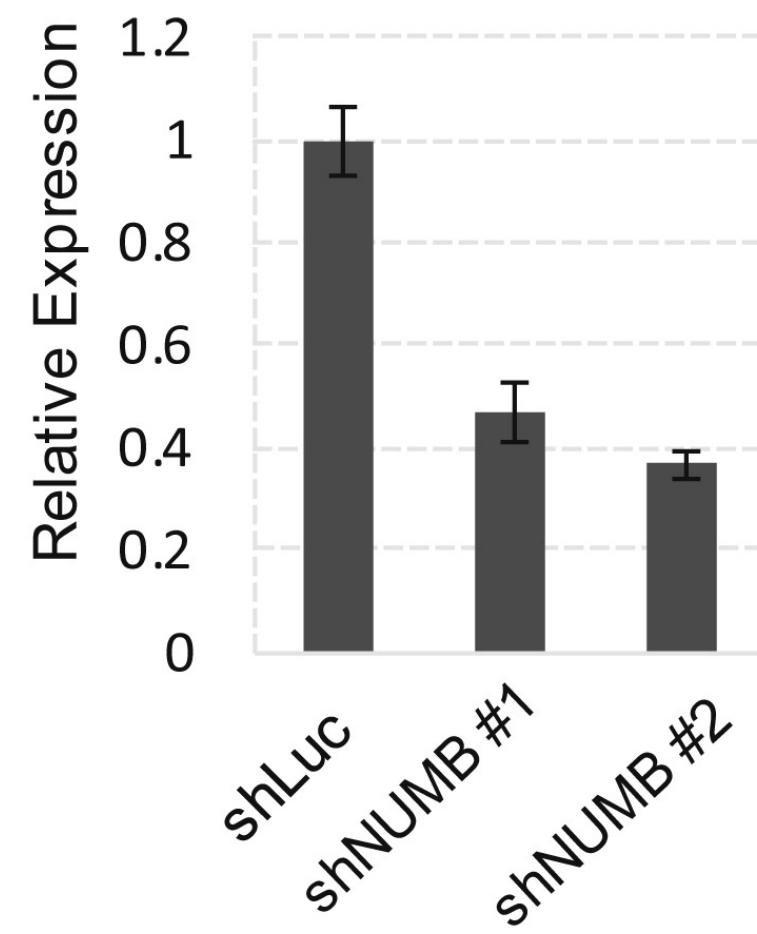WM3451 *NUMB*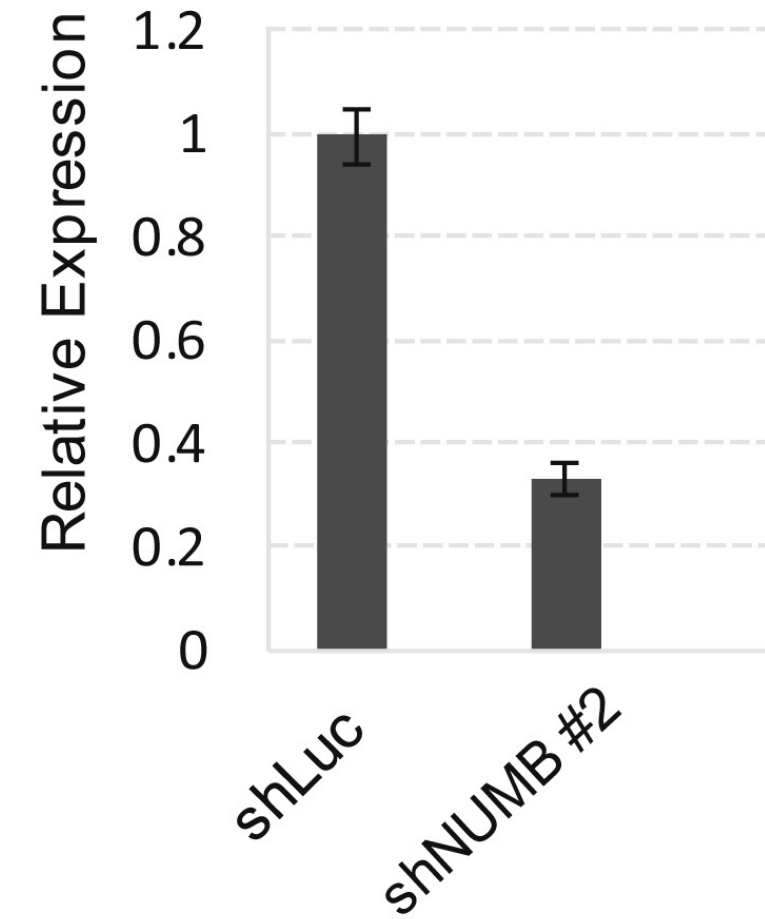**b**

WM1799

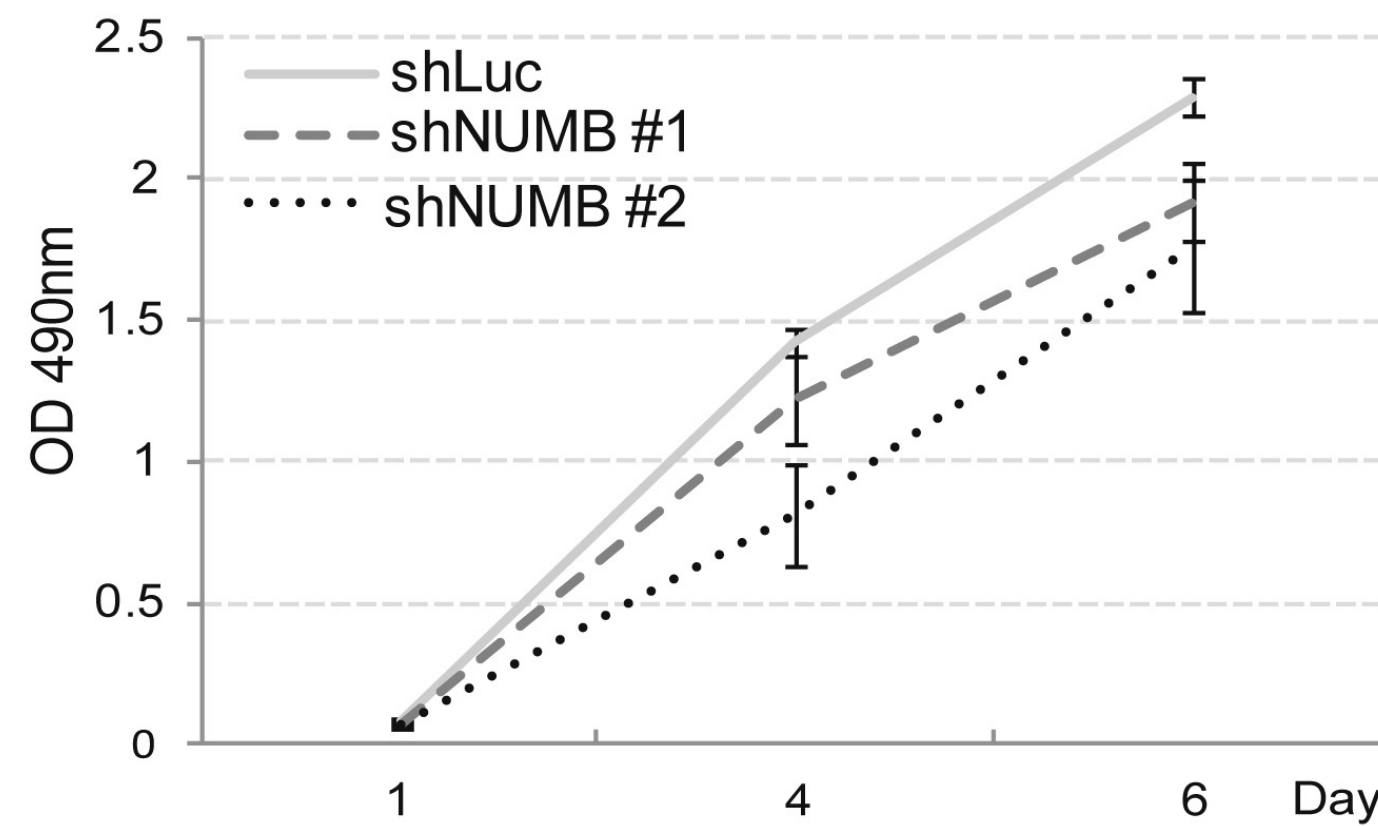

WM3451

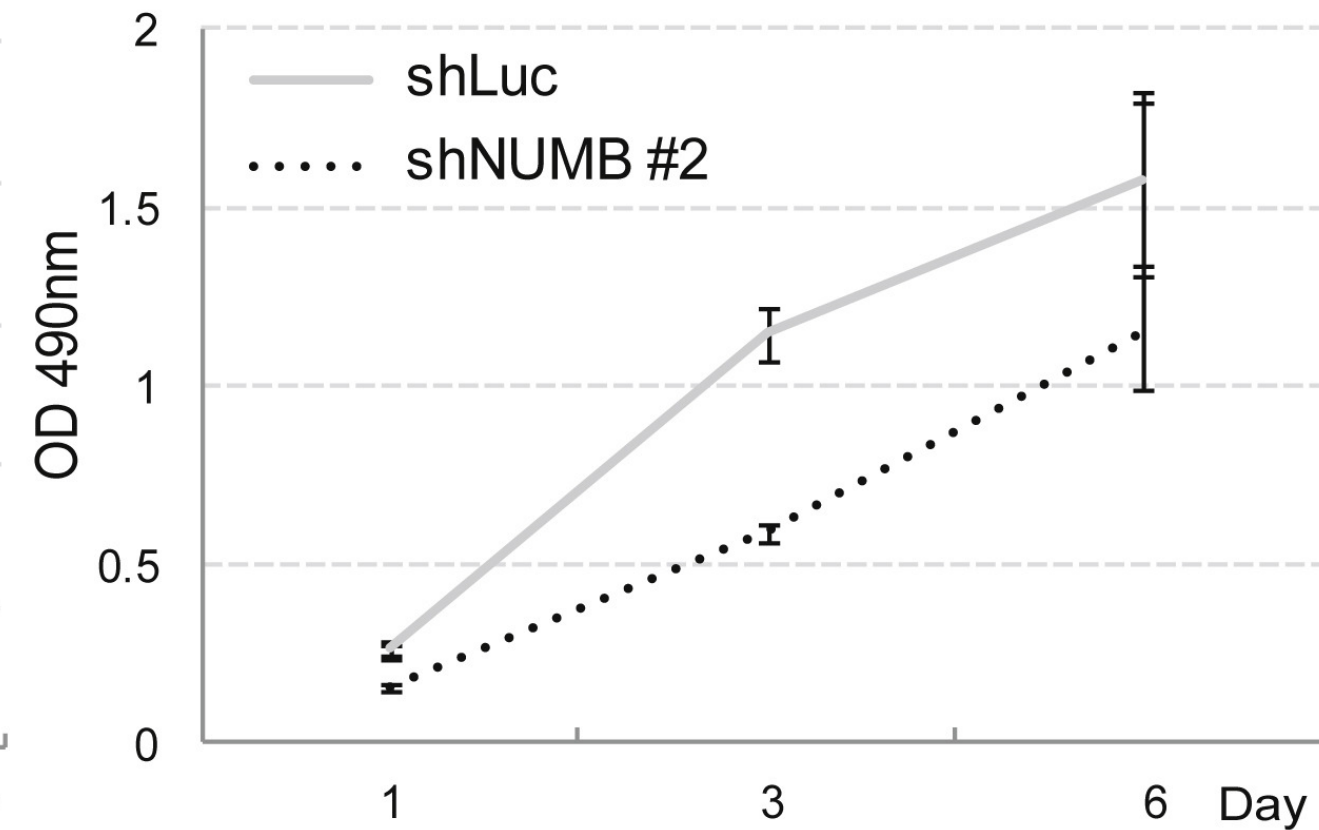

Supplement: Figure S1 [file NIHMS1823153-supplement-Figure_S1.pdf]

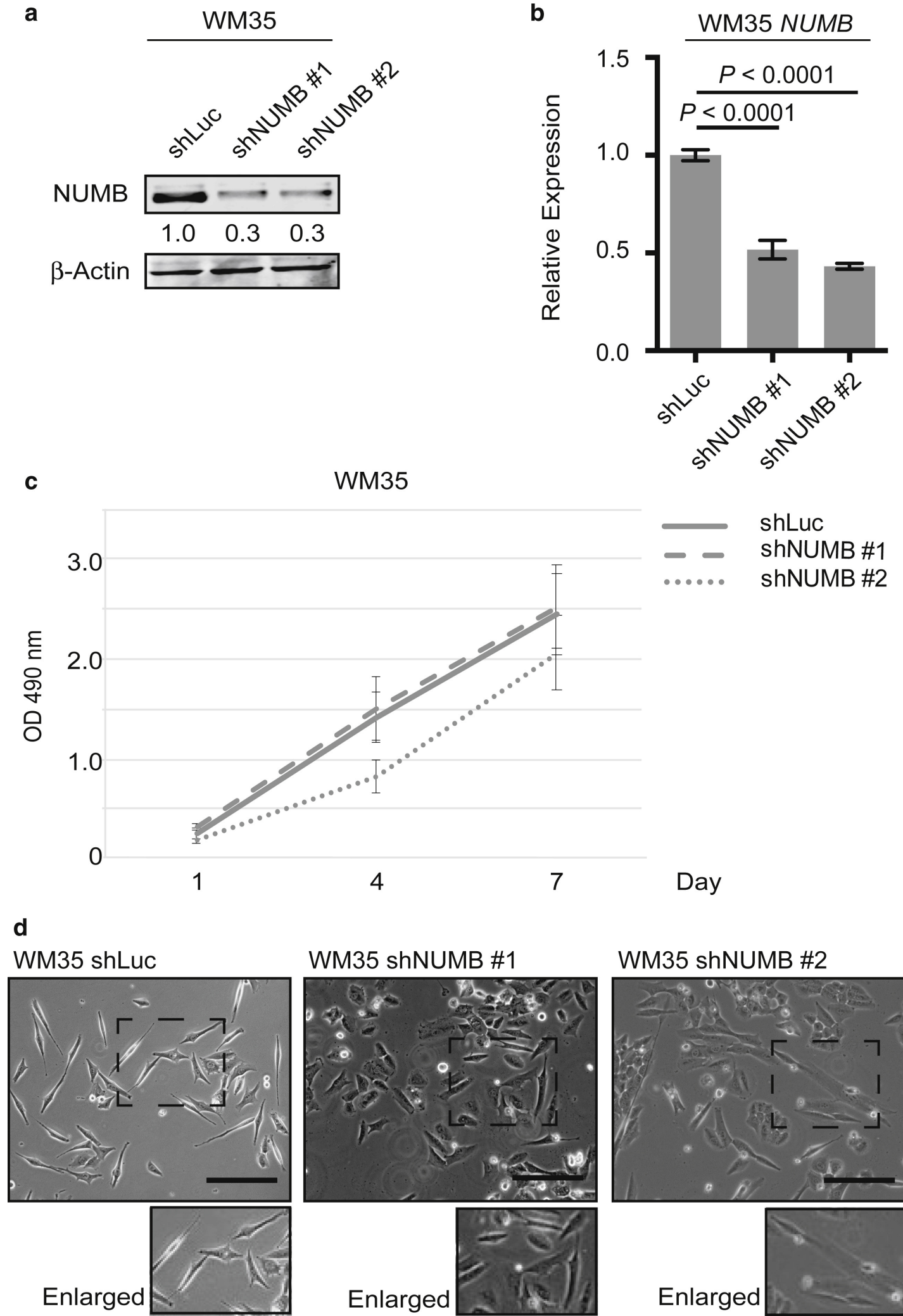

Supplement: Figure S2 [file NIHMS1823153-supplement-Figure_S2.pdf]

**a**

WM1799 shLuc

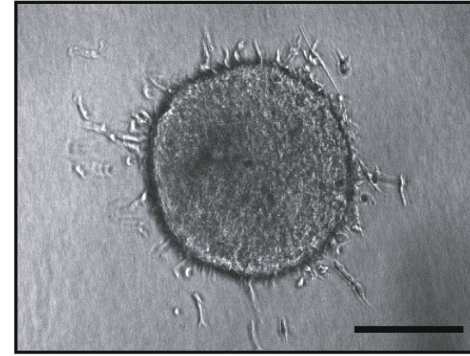

WM1799 shNUMB #1

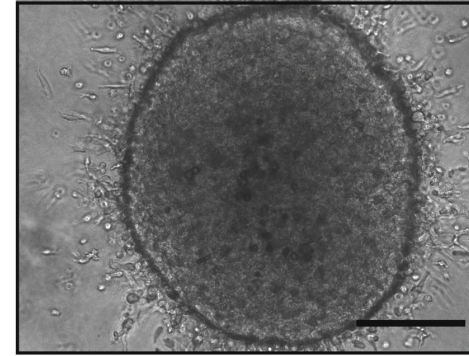

WM1799 shNUMB #2

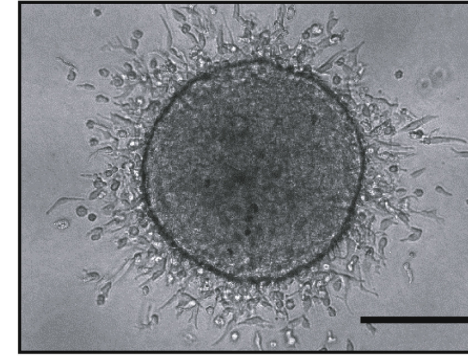**b**

WM3451 shLuc

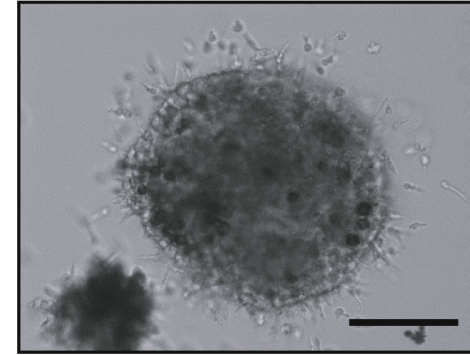

WM3451 shNUMB #2

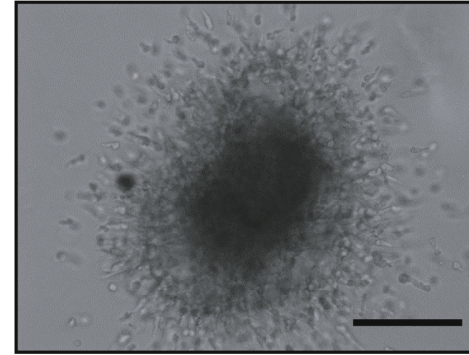**c**

WM35 shLuc

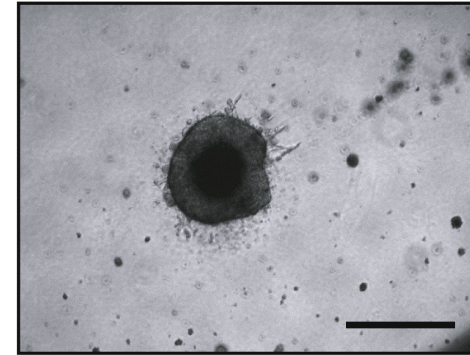

WM35 shNUMB #1

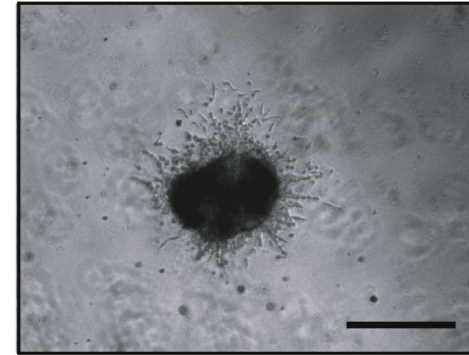

WM35 shNUMB #2

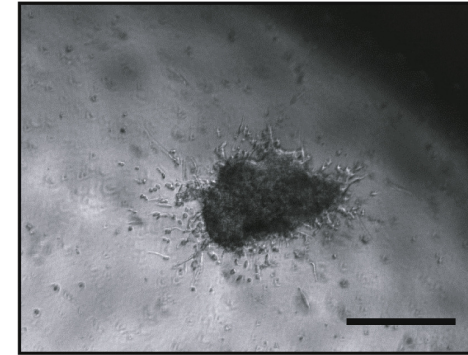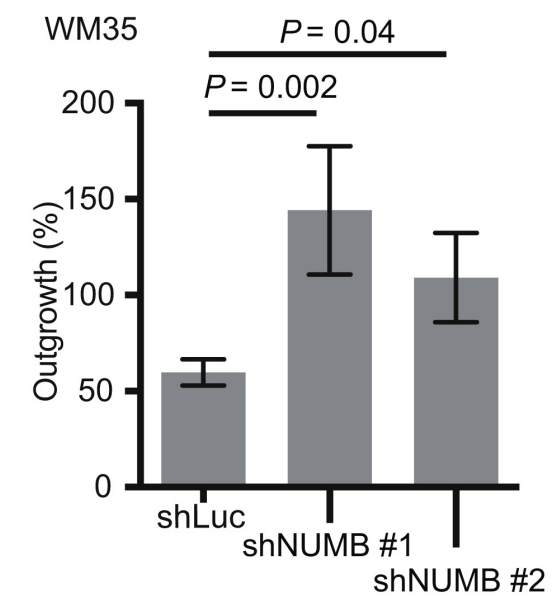

Supplement: Figure S3 [file NIHMS1823153-supplement-Figure_S3.pdf]

WM1799

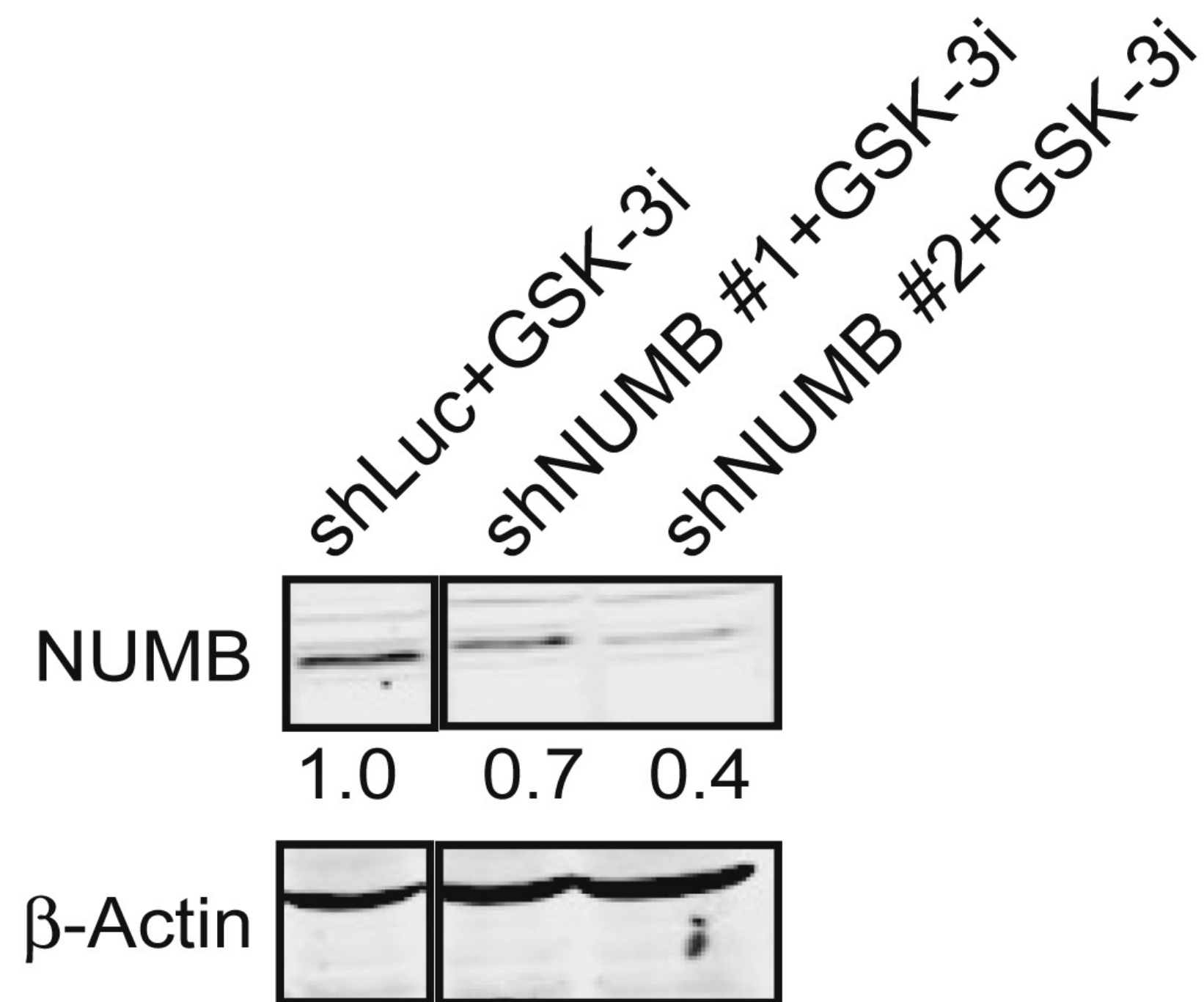

WM1799 *NUMB*

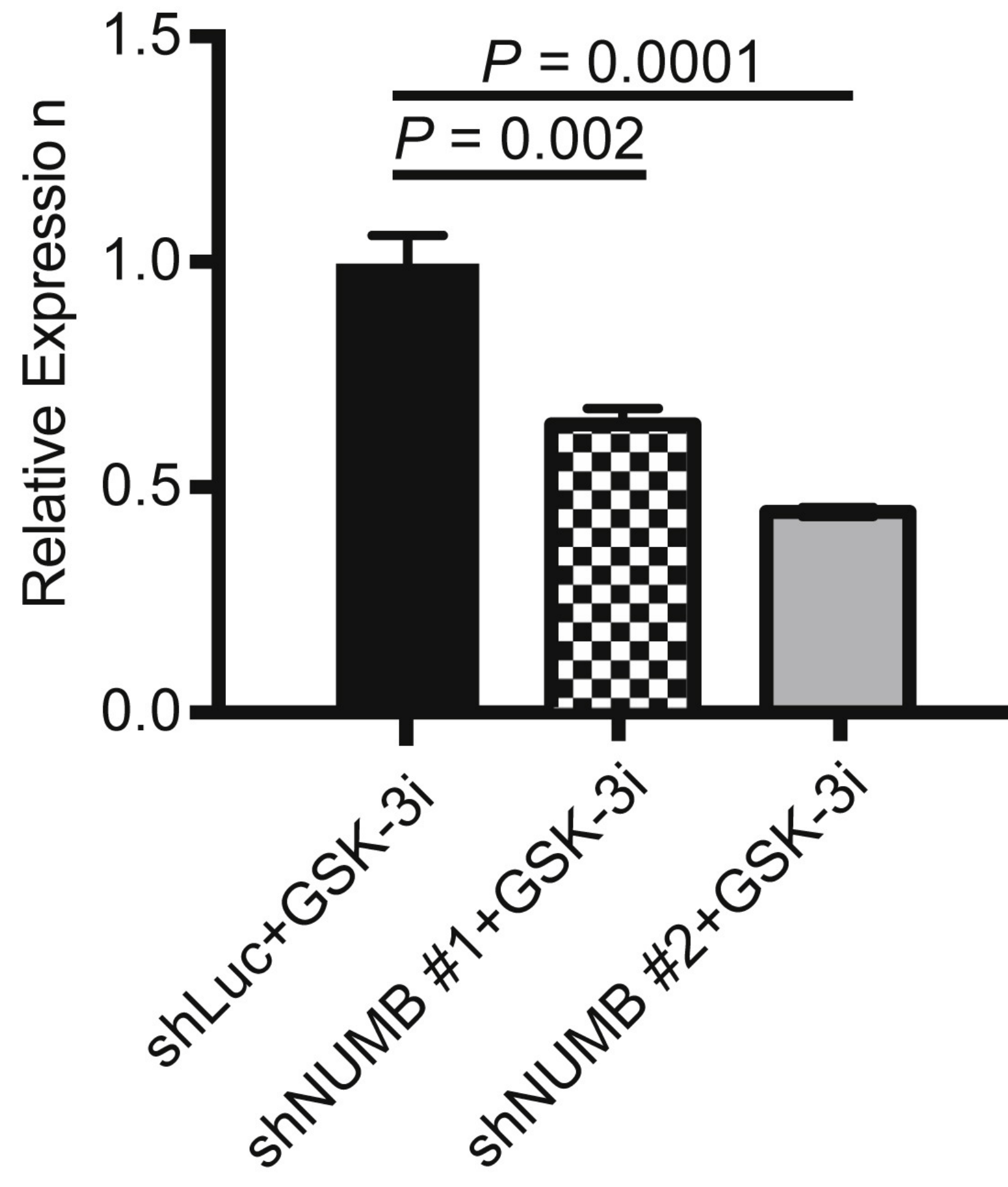

Supplement: Figure S5 [file NIHMS1823153-supplement-Figure_S5.pdf]

WM1799 *AXIN2*

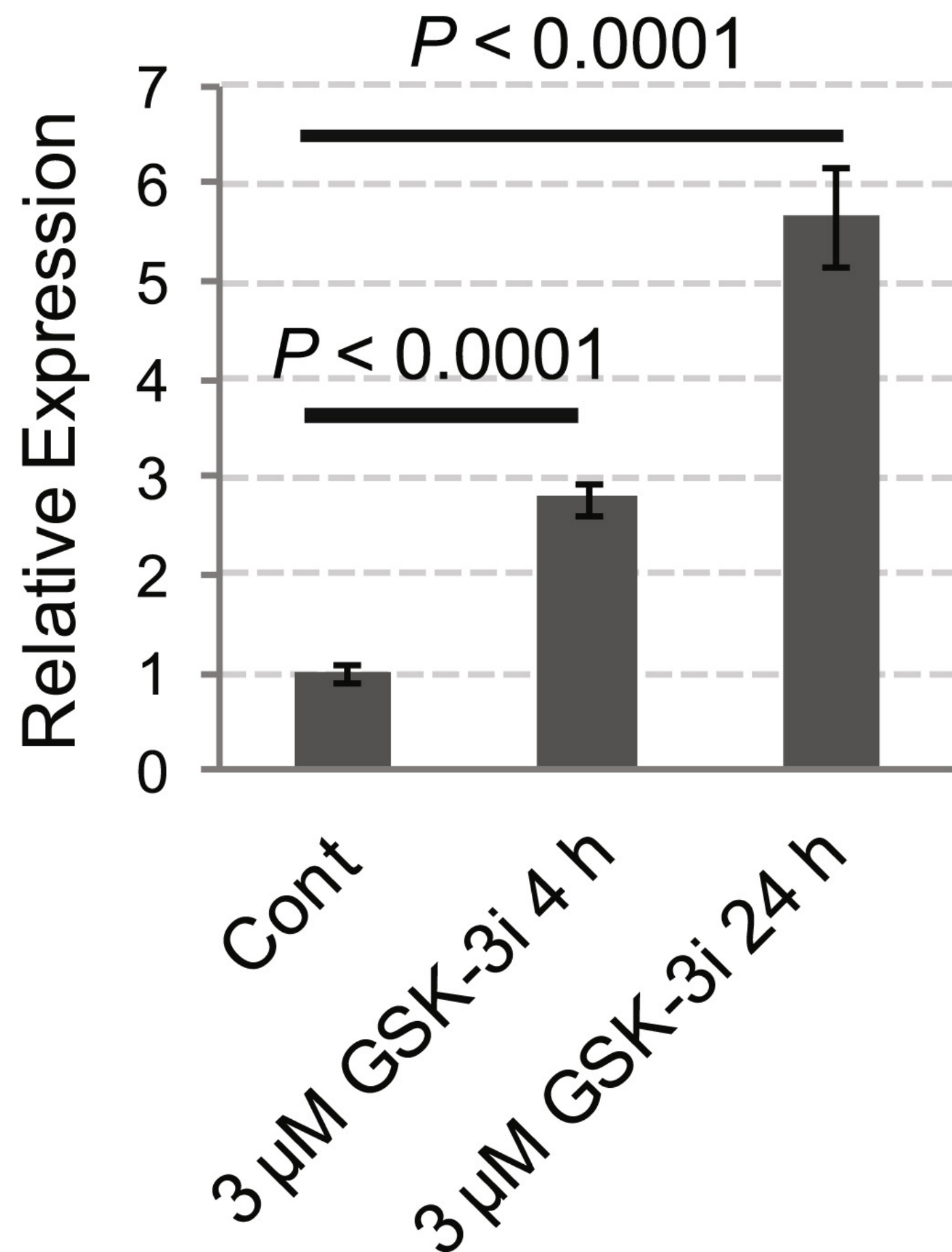

WM3451 *AXIN2*

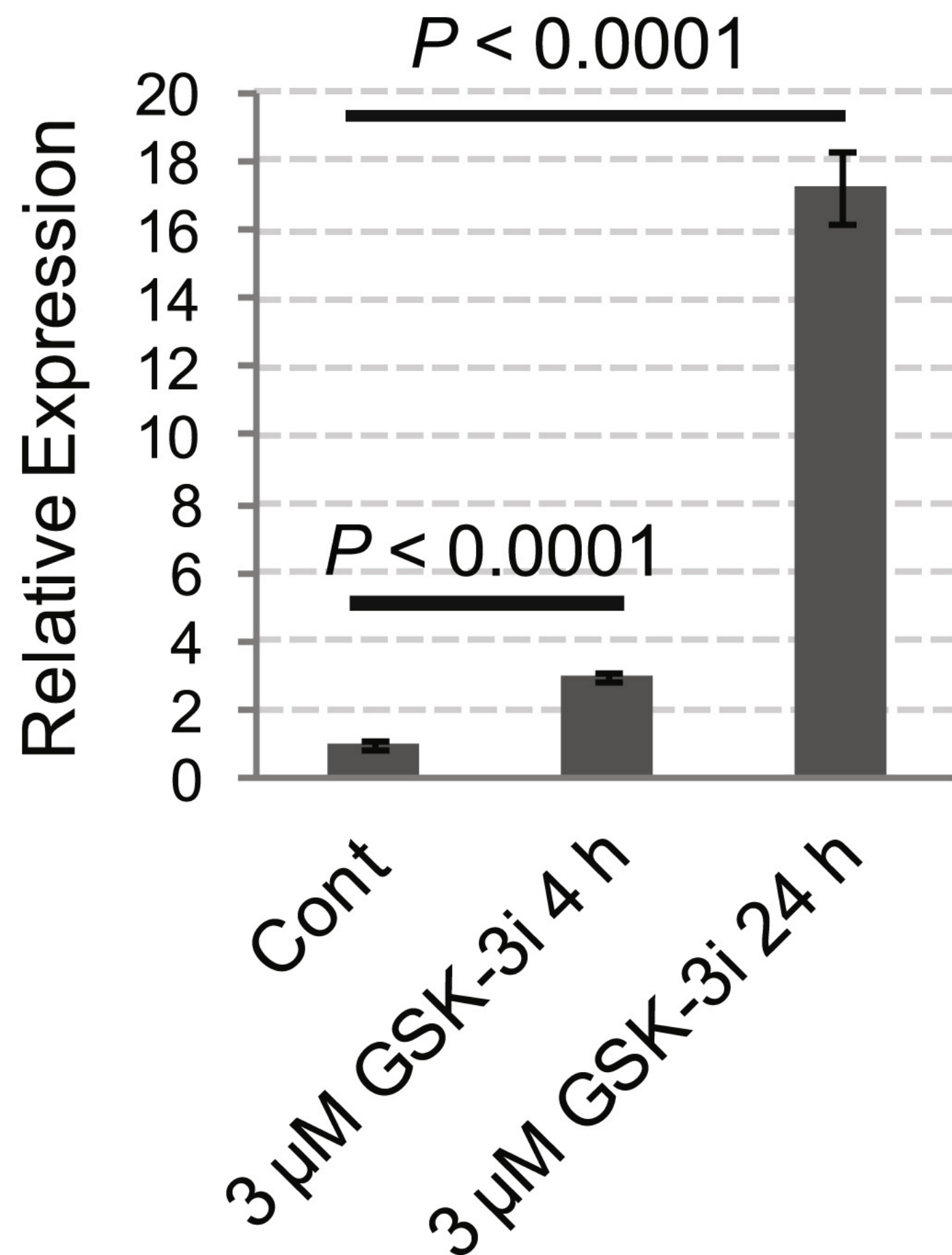

Supplement: Figure S4 [file NIHMS1823153-supplement-Figure_S4.pdf]

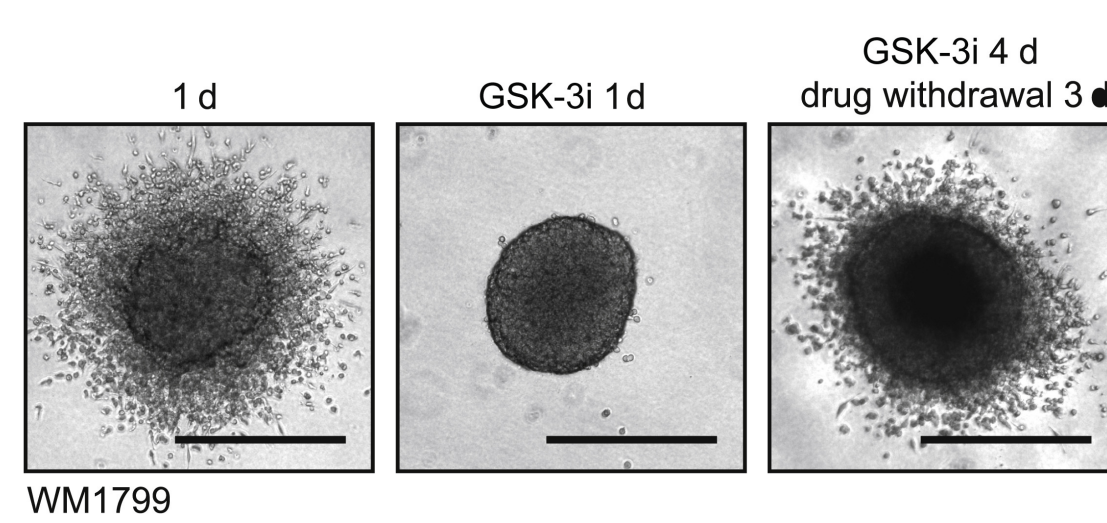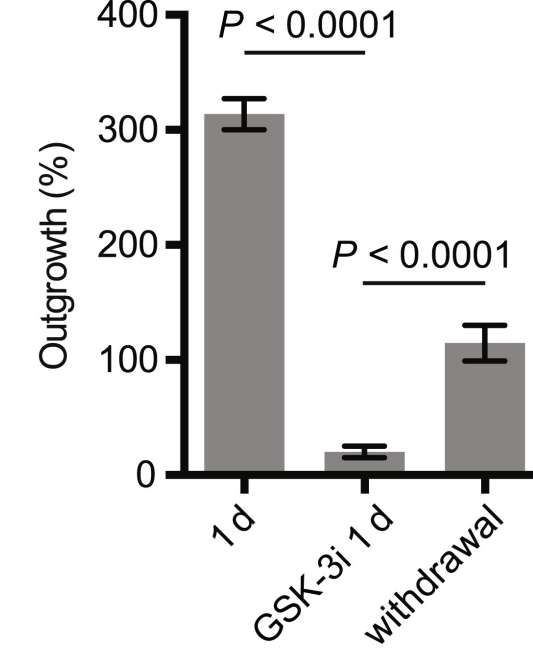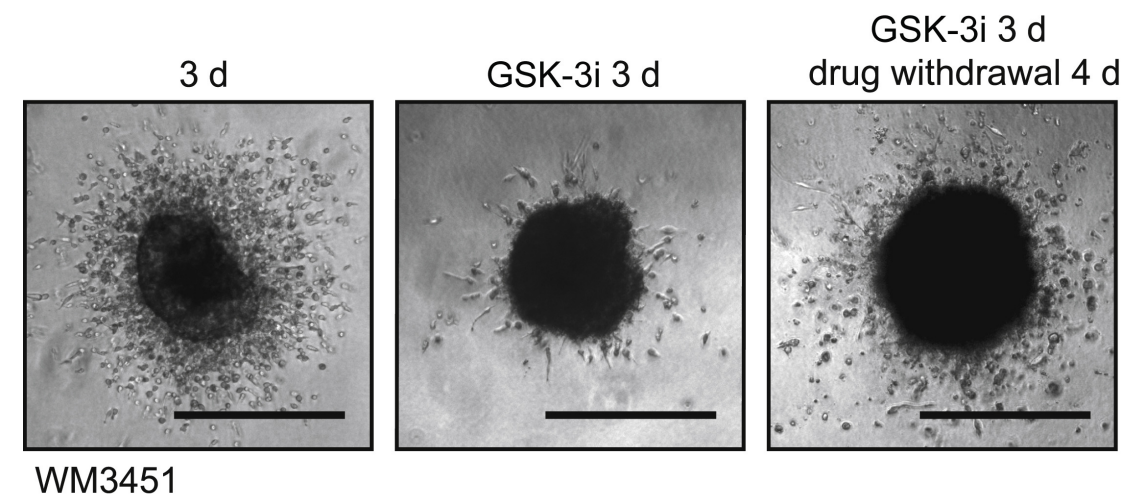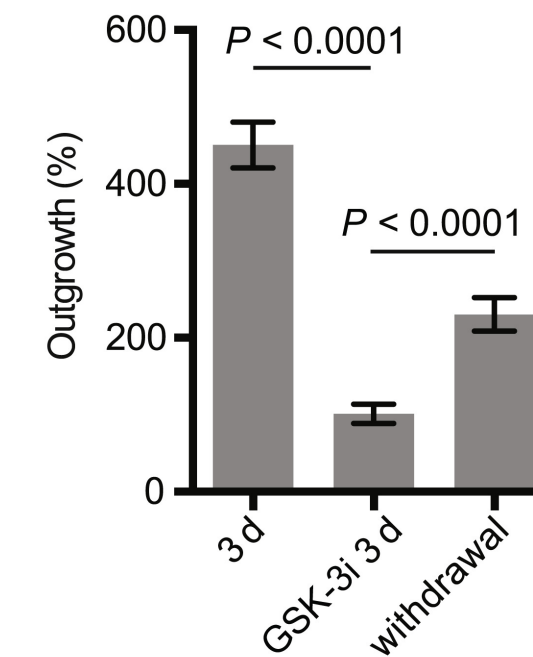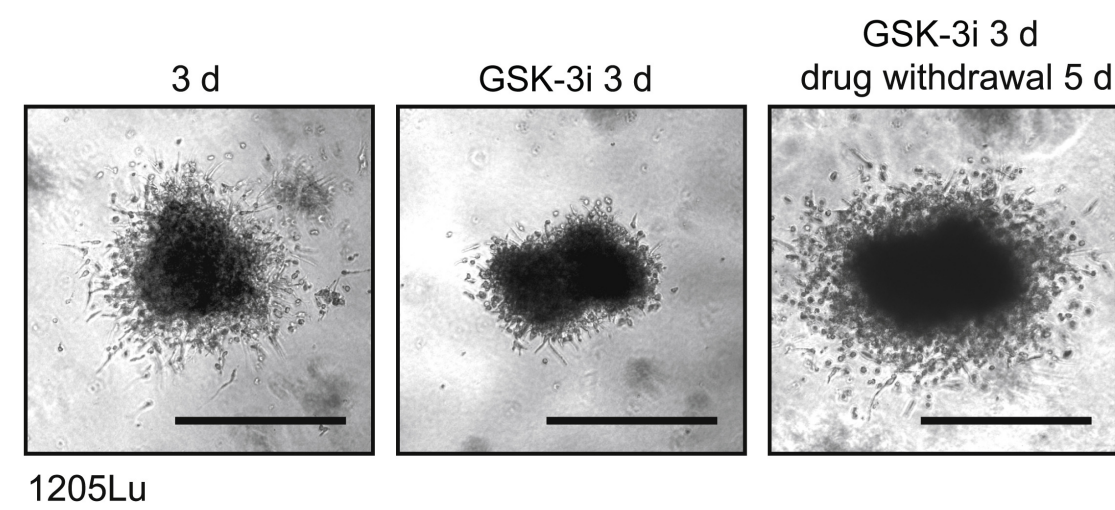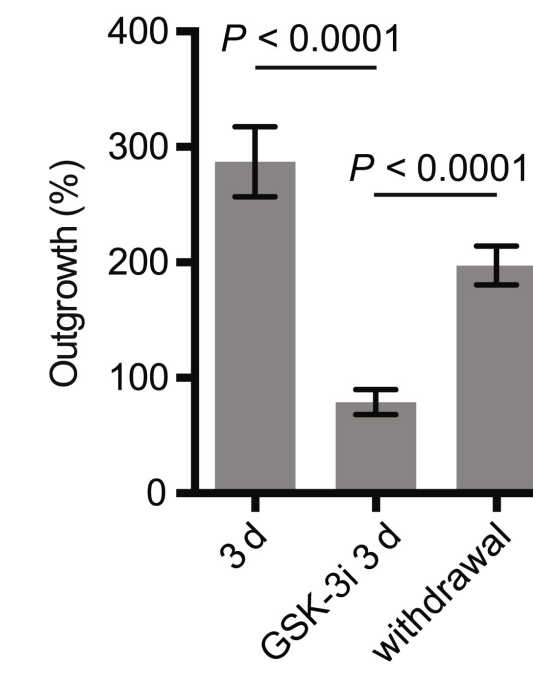

Supplement: Figure S6 [file NIHMS1823153-supplement-Figure_S6.pdf]
