# Supplementary material for: NUMB as a Therapeutic Target for Melanoma
Source: J Invest Dermatol. Author manuscript; Available in PMC 2022 Nov 28. (PMC9704357; doi:10.1016/j.jid.2021.11.027)
Supplement: Suppl Figure Legend [file NIHMS1823153-supplement-Suppl_Figure_Legend.docx]

**Supplementary Figure Legends**

Supplementary Figure S1.

Knockdown efficacy of shNUMB in metastatic melanoma cell lines. (a) Quantitative real time-PCR showing decreased NUMB mRNA levels in the metastatic melanoma cell lines WM1799 and WM3451 transduced with shNUMB #1 and #2 compared with that in shLuc. mRNA levels of

NUMB were normalized to that of GAPDH. (b) The MTS assay was used to assess the effect of NUMB knockdown on cell proliferation. OD, optical density; shLuc, short hairpin RNA targeting firefly luciferase; shNUMB, short hairpin RNA targeting NUMB.

Supplementary Figure S2.

Knockdown efficacy of shNUMB in a primary melanoma cell line. (a) Immunoblot analysis showing NUMB expression in a primary melanoma cell line with a control vector (shLuc) or shNUMB #1 and #2. Blotting for bactin serves as a loading control. The relative intensities of the immunoblot bands were quantified. (b) Quantitative real time-PCR showing decreased NUMB mRNA levels in the primary melanoma cell line WM35 transduced with shNUMB #1 and #2 compared with that in shLuc. mRNA levels of NUMB were normalized to that of GAPDH. (c) The MTS assay was used to assess the effect of NUMB knockdown on cell proliferation. (d) Downregulation of NUMB by shRNA in primary melanoma cell lines leads to morphological changes. Bars = 200 mm. OD, optical density; shLuc, short hairpin RNA targeting firefly luciferase; shNUMB, short hairpin RNA targeting NUMB; shRNA, short hairpin RNA.

Supplementary Figure S3.

Knockdown efficacy of shNUMB in a primary melanoma cell line. (a‒c) shNUMB-transduced cells or control vector‒transduced cells were grown as spheroids embedded in a 3D collagen matrix to test their invasive ability. NUMB-knockdown cell lines showed increased invasion in (a) WM1799 (on day 1) and (b) WM3451 (on day 2) and (c) WM35 (on day 4) melanoma cell lines compared with that in the control cell lines. Bars = 200 mm. 3D, three-dimensional; shLuc, short hairpin RNA targeting firefly luciferase; shNUMB, short hairpin RNA targeting NUMB.

Supplementary Figure S4.

AXIN2 expression in GSK-3i‒treated melanoma cells. Quantitative real time-PCR showing the time-course expression of AXIN2, a target gene of b-catenin, in WM1799 and WM3451 melanoma cell lines treated with GSK-3i IX at 3 mM. The mRNA levels were normalized to that of GAPDH. Cont, no treatment with GSK-3i; GSK-3i, glycogen synthase kinase-3 inhibitor; h, hour.

Supplementary Figure S5.

The rescue efficacy of NUMB knockdown on GSK-3i‒treated melanoma cells. (a) Immunoblot analysis showing NUMB expression in GSK-3 inhibitor‒treated WM1799 with a control vector (shLuc) or shNUMB #1 and #2. Blotting for ß-actin serves as a loading control. The relative intensities of the immunoblot bands were quantified. (b) Quantitative real time-PCR showing decreased NUMB mRNA levels in GSK-3i‒treated WM1799 transduced with shNUMB #1 and #2 compared with that in shLucs. mRNA levels of NUMB were normalized to that of GAPDH. GSK-3i, glycogen synthase kinase-3 inhibitor; shLuc, short hairpin RNA targeting firefly luciferase; shNUMB, short hairpin RNA targeting NUMB.

Supplementary Figure S6. Inhibition of GSK-3 reduces cell invasion in melanoma cell lines. Melanoma cell lines were grown as spheroids embedded in the collagen matrix and were allowed to invade with or without the presence of GSK-3i IX at 3 mM for 1 d or 3 d in WM1799, WM3451, and 1205Lu melanoma cell lines. Inhibition of GSK-3 resulted in delayed cell invasion at the timepoints of 1 or 3 d. After the drug was withdrawn, cells started to invade the collagen matrix. Bars = 200 mm. d, day; GSK-3i, glycogen synthase kinase-3 inhibitor.
